# Race-related differences in antibody responses to the inactivated influenza vaccine are linked to distinct pre-vaccination gene expression profiles in blood

**DOI:** 10.18632/oncotarget.11704

**Published:** 2016-08-30

**Authors:** Raj Kurupati, Andrew Kossenkov, Larissa Haut, Senthil Kannan, Zhiquan Xiang, Yan Li, Susan Doyle, Qin Liu, Kenneth Schmader, Louise Showe, Hildegund Ertl

**Affiliations:** ^1^ The Wistar Institute, Philadelphia, PA, USA; ^2^ Biomedical Graduate Group, University of Pennsylvania, Philadelphia, PA, USA; ^3^ Development and Division of Geriatrics, GRECC, Durham VA Medical Center and Center for the Study of Aging and Human, Department of Medicine, Duke University Medical Center, Durham, NC, USA

**Keywords:** B cell responses, inactivated influenza vaccine, race, Immunology and Microbiology Section, Immune response, Immunity

## Abstract

We conducted a 5-year study analyzing antibody and B cell responses to the influenza A virus components of the inactivated influenza vaccine, trivalent (IIV3) or quadrivalent (IIV4) in younger (aged 35-45) and aged (≥65 years of age) Caucasian and African American individuals. Antibody titers to the two influenza A virus strains, distribution of circulating B cell subsets and the blood transcriptome were tested at baseline and after vaccination while expression of immunoregulatory markers on B cells were analyzed at baseline. African Americans mounted higher virus neutralizing and IgG antibody responses to the H1N1 component of IIV3 or 4 compared to Caucasians. African Americans had higher levels of circulating B cell subsets compared to Caucasians. Expression of two co-regulators, i.e., programmed death (PD)-1 and the B and T cell attenuator (BTLA) were differentially expressed in the two cohorts. Race-related differences were caused by samples from younger African Americans, while results obtained with samples of aged African Americans were similar to those of aged Caucasians. Gene expression profiling by Illumina arrays revealed highly significant differences in 1368 probes at baseline between Caucasians and African Americans although samples from both cohorts showed comparable changes in transcriptome following vaccination. Genes differently expressed between samples from African Americans and Caucasians regardless of age were enriched for myeloid genes, while the transcripts that differed in expression between younger African Americans and younger Caucasians were enriched for those specific for B-cells.

## INTRODUCTION

The efficacy and immunogenicity of vaccines varies depending on the study cohort. Race and ethnicity were shown to affect antibody responses to the rubella vaccine, which elicited significantly higher titers in children of African ethnicity compared to those of European descent or Hispanic ethnicity [1]. A study conducted in the US found significantly higher seroprevalence rates of antibodies to measles virus in African Americans compared to Caucasians [2] and antibody titers to the pertussis vaccine were strongly and consistently higher in African American children compared to Caucasian children [3]. A similar study conducted in Northern Canada showed that native Innuit and Innu infants developed higher antibody titers to a measles vaccine as compared to those of Caucasian descent [4]. Disparities in serologic responses to vaccines were also observed between different ethnic groups for the *Haemophilus influenzae* type b-tetanus toxoid conjugate vaccine [5], or the *Haemophilus influenzae* type b polysaccharide-*Neisseria meningitidis* outer membrane protein conjugate vaccine [6]. There is thus ample evidence that ethnicity affects responsiveness to a vaccine.

Other factors such as geography play a role. Bacillus Calmette-Guérin (BCG), the only licensed vaccine to prevent tuberculosis, is associated with better vaccine efficacy at a greater distance from the equator [7]. RotaTeq, a commercially available vaccine against rotavirus, showed distinct patterns of efficacy in various regions. Efficacy against hospitalizations and emergency department visits was 97% in the US, 95% in Europe, 90% in Latin America/Caribbic [8] but only 48.3% in Asia and 39.3% in Sub Saharan Africa [9]. Duration of protection differed and was more sustained in Asia than Africa. The causes of these differences are unknown.

Age affects an individual's ability to mount immune responses to vaccines [10] as has been repeatedly demonstrated for influenza vaccines, which on average show 80-90% efficacy in younger populations but only 30-50% in the aged in preventing complications from influenza infections [11]. Defects in both innate and adaptive responses accumulate during aging, a phenomenon referred to as immunosenescence. The output of naïve cells of the adaptive immune system declines [11], T and B cell repertoires become more restricted [12, 13], CD4^+^ T cells loose the ability to provide appropriate help for differentiation of B cells into antibody secreting cells (ASCs) [14] and B cells become more prone to differentiated into short-lived plasma cells upon stimulation rather than undergo germinal center maturation [15], which is required for antibody class switching and affinity maturation.

We conducted a 5-year study analyzing antibody and B cell responses to the influenza A virus components of IIV3 or 4. Younger (aged 30-40) and aged (≥65 years of age) Caucasian and African American individuals were enrolled. Blood was collected before and after IIV3 or 4 vaccination to determine changes in antibody titers, distribution of circulating B cell subsets and expression of immunoregulatory markers on B cells. In addition, the blood transcriptome was analyzed at baseline and at day 7 after IIV3 or 4 vaccination for years 2-5 of the study. African Americans mounted higher virus neutralizing antibody responses to the H1N1 component of IIV3 or 4 when compared to Caucasians. They also mounted higher IgG responses to H1N1 and there was a trend towards higher IgG responses to H3N2. At baseline African Americans had higher levels of circulating B cells compared to Caucasians and this difference was significant for most B cell subsets. In addition, two co-regulators, i.e., programmed death (PD)-1 and the B and T cell attenuator (BTLA) were differentially expressed on B cells of the two cohorts. Taking age into account these differences were seen between younger African Americans and younger Caucasians while results obtained with samples of aged African Americans were similar to those of aged Caucasians. Gene expression profiling by Illumina arrays revealed highly significant differences in 1368 probes at baseline between Caucasians and African Americans although both cohorts showed comparable changes following vaccination.

## RESULTS

### Cohorts and study design

A total of 59 younger (age 30-40) and 80 aged (≥65 years of age) human subjects were enrolled over a 5-year period starting in fall of 2011 and ending in fall of 2015 (Suppl. Table 1A). A number of individuals participated repeatedly (Suppl. Table 1B) so that a total of 115 matched samples from younger and 165 matched samples from aged individuals were analyzed. Of these 270 samples, 27 were from African Americans, 246 from Caucasians, and the remaining 6 from American Indians, Alaskan Native People, Asians or individuals of mixed race. When samples were stratified according to age, there was a bias of higher enrollment of African Americans into the younger (17 samples from African Americans, 95 samples from Caucasians) than the aged cohort (10 samples from African Americans, 150 samples from Caucasians, p-value by Fisher's exact test = 0.022). The average age of African Americans was 36 and 75 for the young and aged cohorts, respectively, while the Caucasians were on average 35 and 76 years of age, respectively (Table 1). In all cohorts regardless of ethnicity or age more females than males participated (Table 1). There were also clear differences in reporting of previous influenza vaccination (Table 1). While the majority of individuals reported that they had been vaccinated during the last 5 years, only 6 percent of younger African Americans had received annual vaccinations while this number increased to 90% in the aged African Americans. The age-related difference in annual Influenza vaccination was less pronounced for Caucasians; 42% of younger Caucasians had received an Influenza vaccine annually compared to 57% of the aged individuals in this group.

**Table 1:** 

Cohort	Average Age	% of total		
		Gender % Females	Flu Vaccination History	
			Ever	Annual
AA, Y	36	59	65	6
AA, A	75	70	100	90
C, Y	35	69	84	42
C, A	76	67	67	57

Blood was collected at baseline just prior to vaccination with IIV3 or IIV4. All individuals received the regular dose of IIV3 or 4, which is approved for use in the young and the aged. The second and third blood samples were collected on days 7 and 14 or 21 after vaccination. Serum samples from all three time points were tested for neutralizing antibodies to the two influenza A viruses, i.e., H1N1 and H3N2. Sera collected at baseline and after vaccination were also tested for H1N1 and H3N2-specific antibodies by ELISA to determine antibody isotypes. Peripheral blood mononuclear cells (PBMCs) collected before and after vaccination were analyzed by flow cytometry to determine numbers and percentages of circulating B cells belonging to different subsets and to determine levels of expression of additional surface markers at baseline. Blood collected into PaxGene tubes at baseline and on day 7 after vaccination was used for RNA isolation to determine differences in the transcriptome. Data were compared between Caucasians and African American samples regardless of age and then stratified according to age groups.

### Virus neutralizing antibody responses

**Figure 1:**
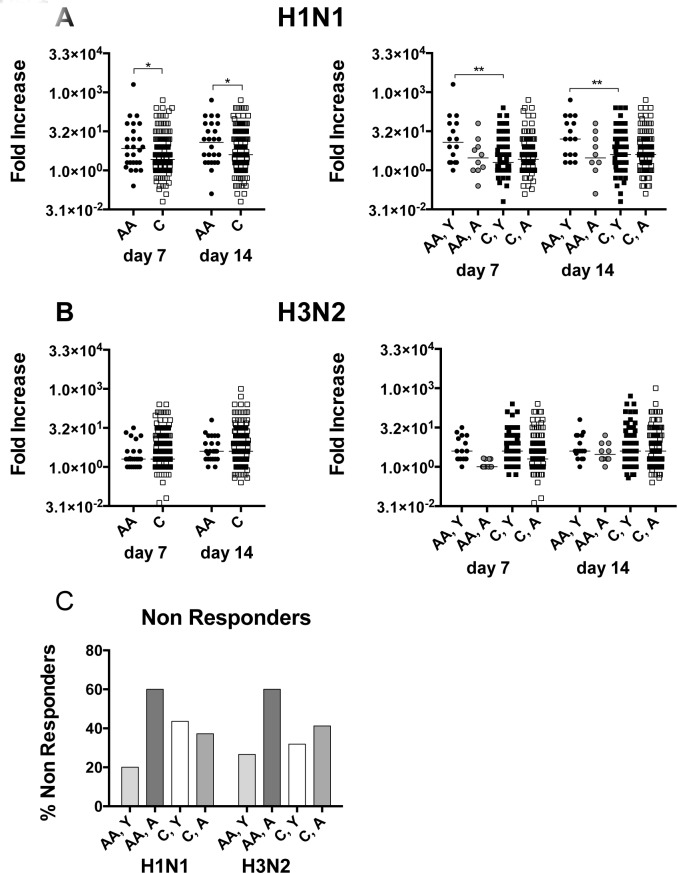
Virus neutralizing antibody responses **A.** shows fold increases in neutralizing antibody titers to H1N1 virus for individual samples with lines indicating medians. Data are compared between African Americans (AA, dark grey bars) and Caucasians (C, light grey bars). The left graph compares African Americans and Caucasians of the entire cohort; the right graph compares younger African Americans to younger Caucasians and aged African Americans to aged Caucasians. Line with stars above indicate significant differences by Wilcoxon rank-sum test, *p ≤ 0.05, **p ≤ 0.01. **B.** Organized in the same fashion as A shows fold increases to H3N2 virus. **C.** shows percentages of non responders to H1N1 and H3N2 virus defined as individuals that either failed to show titer increases equal or above 4 fold over baseline or that failed to developed titers equal or above 1:40. Number of samples per group: AA: 26, C: 244, AA, Y: 16, AA, A: 10, C, Y: 95, C, A: 149.

We initially compared virus neutralizing antibody titers to H1N1, a virus that remained the same during the 5-year study period, and H3N2, which was updated several times, at baseline and following vaccination. Each year the virus strain present in the vaccine was used for serogical assays. We calculated increases in antibody titers as the ratio of titers pre- and post-vaccination and percentages of responders that increased titers by at least 4 to ≥1:40. Some individuals were enrolled only once by our group; others participated repeatedly. When we compare antibody increases after vaccination we find no significant differences between younger or aged individuals that participated once or repeatedly (data not shown). Although there are no differences in absolute antibody titers at the different time points between samples from Caucasians and African Americans (not shown), the former group show significantly higher increase in titers to H1N1 virus (Figure 1A) while responses to H3N2 virus are similar (Figure 1B). Differences in responses to H1N1 are driven by results from samples from younger African Americans, who have significantly higher increases in antibody titers to H1N1 compared to younger Caucasians. Comparison rates of non-responders, defined as individuals that fail to show a 4-fold increase in titers at either post-vaccination time point and reached titers of at least 1:40, are comparable between younger and aged Caucasians while there is a very marked difference in non-responder rates to both influenza A virus stains between younger and aged African Americans (Figure 1C). Titers of antibodies to H1N1 and H3N2 stratified according to year of vaccination are furthermore shown for the younger and aged cohorts in Suppl. Figure 1. There are some differences in baseline titers or titers after vaccination to H1N1 and H2N3 in the different years but responses measured as fold increases over baseline are in most years comparable.

### IgG and IgM responses

**Figure 2:**
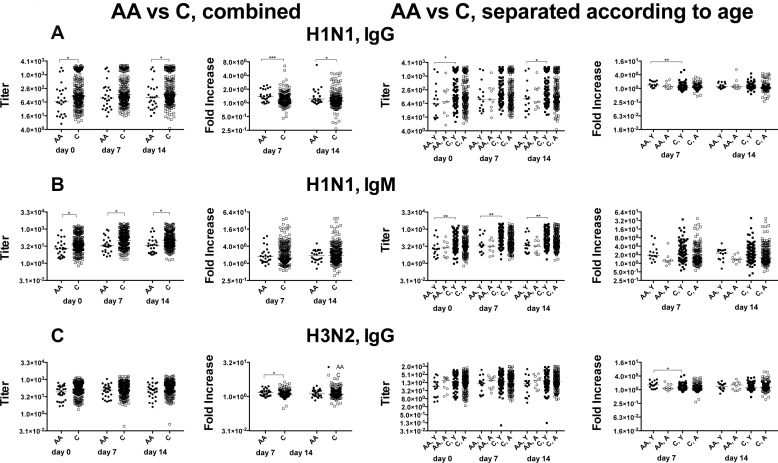
IgG and IgM antibody responses A shows to the left IgG antibody titers to H1N1 virus in African Americans and Caucasians followed by fold increases. The next graph shows the same data sets separating results from younger and aged individuals. Data are shown for individual samples with lines indicating medians. Line with stars above indicate significant differences by Wilcoxon rank-sum test, *p ≤ 0.05, **p ≤ 0.01, *** p ≤ 0.001. **B.** and **C.** organized in the same fashion as **A.** shows data for H1N1-specific IgM responses and H3N2-specific IgG titers and responses respectively. Number of samples per group: AA: 25, C: 243, AA, Y: 16, AA, A: 9, C, Y: 94, C, A: 149.

To further test overall antibody responses, sera were tested by ELISA for titers of IgA, IgG and IgM to the two influenza A virus strains. IgA responses are comparable between the cohorts (data not shown). African Americans have lower H1N1 IgG titers at baseline and on day 14 after vaccination. These differences are driven by samples from younger African Americans, who unlike the aged show significant differences to the age-matched cohort of Caucasians (Figure 2A). Increases in H1N1 IgG titers following vaccination are higher in African Americans compared to Caucasians and again this is due to better responses of younger African Americans. IgM titers against H1N1 virus are lower in African Americans due to results from the younger cohort (Figure 2B). H3N2 IgG titers are comparable between the cohorts at all 3 time points but younger African Americans mounted higher responses compared to younger Caucasians on day 7 after vaccination (Figure 2C).

### B cell subsets and phenotypes

**Figure 3:**
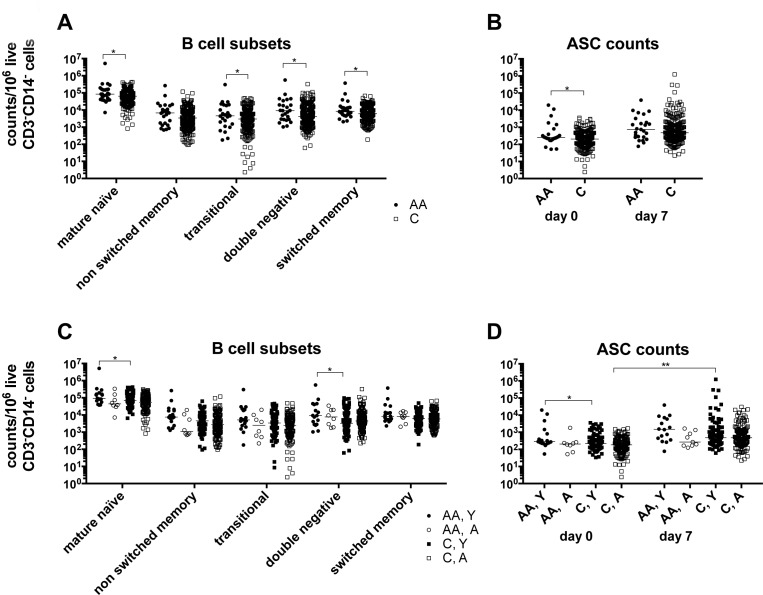
B cell subsets **A.** shows normalized numbers of B cell belonging to different subsets at baseline in African Americans and Caucasians. **B.** shows normalized numbers of ASCs at baseline and on day 7 after vaccination in African Americans and Caucasians. Data are normalized to 10^6^ live CD3^−^CD14^−^ cells and shown for individual samples with medians indicated by the lines. **C.** and **D.** show the same data sets separating results from younger and aged individuals. Line with stars above indicate significant differences by Wilcoxon rank-sum test, * *p* ≤ 0.05, ** *p* ≤ 0.01. Number of samples per group: AA: 25, C: 235, AA, Y: 17, AA, A: 8, C, Y: 92, C, A: 143.

At baseline we determined in about half of the subjects white blood cell counts. There are no significant differences in the distribution of neutrophils, lymphocytes, monocytes, eosinophiles or basophiles comparing African Americans and Caucasians of the two age groups. Within lymphocytes, percentages of B cells, identified by stains for CD19 are also comparable (Suppl. Figure 2). We used cellular stains with B cell-defining antibodies to CD19, IgD, CD20, CD27 and CD38 followed by multi-color flow cytometry to identify and enumerate cells belonging to different subsets. There is a trend towards higher numbers of circulating B cells in African Americans, with significant differences for mature naïve, transitional, double-negative and switched memory B cells (Figure 3A) and antibody secreting cells (Figure 3B). These differences are caused by samples from younger African Americans, who at baseline have higher numbers of circulating mature naïve, double negative B cells (Figure 3C) and antibody-secreting cells (ASCs) (Figure 3D) compared to younger Caucasians. Nevertheless, significant increases in ASCs compared to baseline after vaccination are only observed on day 7 in the younger Caucasian cohort.

**Figure 4:**
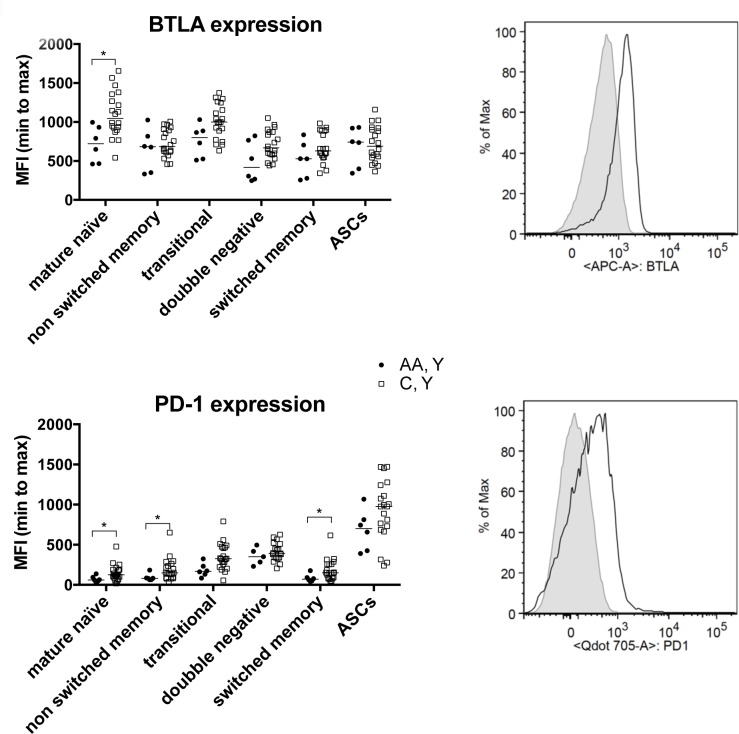
B cell phenotypes The graphs show expression levels of BTLA and PD-1 on different B cell subsets from younger African American and Caucasian individuals. Data are shown for individual samples with the line indicating the median fluorescent intensity. Lines with stars above indicate significant differences by Wilcoxon rank-sum tests were performed. Number of samples per group: AA, Y: 6, AA C, Y: 20. The histograms to the right show flow cytometry data from 1 younger African American (shaded histogram) and one younger Caucasian (open histogram) individual.

We compared the expression of two immunoregulators on B cells, BTLA and PD-1, in the two younger cohorts in responses to the influenza A virus components. There is a trend of higher expression of both markers on B cells from younger Caucasians, which reach significance for BTLA on mature naïve B cells and for PD-1 on mature naïve, and unswitched and switched memory B cells (Figure 4).

### Gene expression profile differences between African Americans and Caucasians

We performed gene expression profiling using Illumina microarrays on blood collected at baseline and on day 7 after vaccination in years 2 to 5. Changes in the transcriptome following vaccination are comparable between Caucasians and African Americans and none of genes show significant differences in response (best FDR=47%). Nevertheless, there are significant differences at baseline between the two cohorts. Specifically, in blood from African Americans, 1368 probes significantly differed with FDR<5% and fold changes of at least 1.2 from those of Caucasians across all 4 years. Significantly different expressed genes are shown in Suppl. Table 2.

Probes that showed race-related differences in both age cohorts include 29 for genes encoding mitochondrial proteins; 23 of those expressed higher in younger and aged African Americans compared to the caucasian cohorts. The 3 differentially expressed transcripts for peroxisomal proteins are lower in African Americans. Of the differentially expressed 12 transcripts for ribosomal proteins 4 are higher in both younger and aged African Americans than younger and aged Caucasians. Several key transporters for nutrients also differ between the two cohorts. Solute carrier family 2, member 1 (SLC2A1, 1.45) is higher in African Americans while solute carrier family 2, member 9 (SLC2A9, -1.27) is lower. Both facilitate transport of glucose. Solute carrier family 27, member 1 (SLC27A1, -1.29), involved in fatty acid transport, is lower in African Americans. Several CD markers show race-specific expression patterns regardless of age. Expression of CD22 (1.22), an inhibitor of B cell receptor signaling is higher in African Americans while expression levels of CD1Dd (−1.24), an antigen-presenting protein for glycolipids, CD14 (−1.31), CD93 (−1.27), CD93 (−1.27), CD163 (−1.32), CD300C (−1.44), which are largely myeloid cell markers, and CD320, which augments proliferation of plasma cell precursors, are lower in African Americans. Expression of genes encoding cytokines or cytokine receptors including IL-8 (−1.63), IL-1R2 (−1.24), IL-6R (−1.31), IL-12RB1 (−1.26), and IL-17RA (−1.35) is reduced in African Americans.

Another pathway that is essential for plasma cell differentiation and function is autophagy,^17^ which maintains cellular metabolism when nutrients become limited. The autophagy-related genes that are differentially expressed in our two racially distinct cohorts, i.e., ATG7, ATG4 and ATG1A LAMP1 and 2 and VPS18 are lower in African American samples.

**Figure 5:**
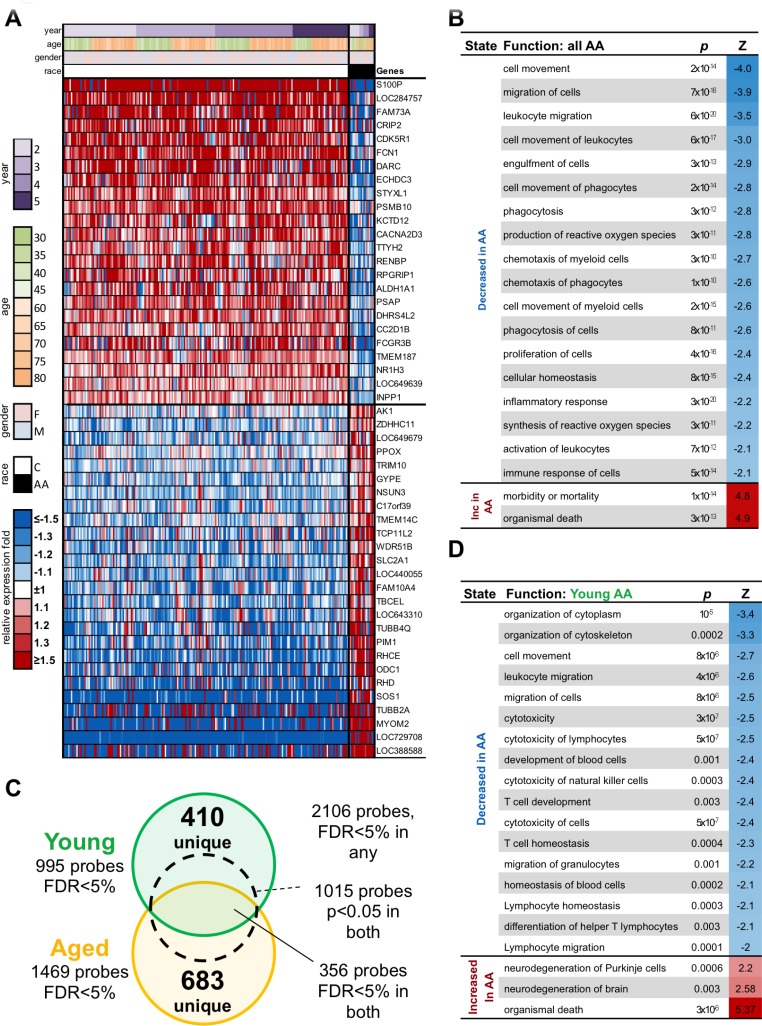
Genes differentially expressed in AA compared to C cohorts **A.** Gene expression heatmap of top 50 probes significantly different between All AA and all C subjects. **B.** Top 20 functions enriched among genes significantly different between AA and C subjects of any age. **C.** Overlap between probes significantly different between AA and C subject within Young and Aged groups. **D.** Top 20 functions enriched among genes uniquely significantly different between AA and C subjects within Young group.

We also compared the two cohorts for enrichment of functional or regulatory categories using Ingenuity Pathway Analysis. Among significantly enriched functions (Figure 5B), inflammatory responses, cell signaling, antigen presentation, scavenging of free radicals, lipid metabolism and cellular movements are decreased in African American samples. Signaling pathways, which are reduced in African Americans, include interferon (IFN) and interleukin (IL)-8 signaling. A number of regulators differentiate the two cohorts; most are reduced in African Americans such as regulation through type 1 (−3) and 2 (−4.2) IFNs, IL-1B (−2.1), IL-2 (−2.3), IL-15 (−2.3), IL-27 (−2.6), Stat3 (−2.1), Toll like receptor (TLR)-7 (−2.6) and CpG oligonucleotides (−2.6). Genes regulated by IL-3 (2.2) or FoxP3 (2.2) are overexpressed in African Americans. A total of 25 networks show differences between the two ethnic groups. Several of those involve metabolic networks, specifically lipid, amino acid and carbohydrate metabolism as well as energy production. Analysis by DAVID software identifies differences in the anabolic pentose phosphate pathway, which is reduced in African Americans. Caucasians show decreases in oxygen transport, oxygen carriers and heme biosynthesis, while lymphocyte proliferation, immune responses, antigen processing and presentation as well as glucose catabolism are higher. We then tested the gene list for enrichment of genes specific to some blood cell type using the IRIS dataset [16]. We find that genes differently expressed between African Americans and Caucasians regardless of age are enriched for myeloid genes (66 genes, 2.5 fold enrichment, p=3×10^−11^, 61 of 66 down-regulated in African Americans).

### Differences in gene expression profile between younger African Americans and younger Caucasians not found among aged African Americans and aged Caucasians

Since differences in antibody responses and B cell subset distribution in blood are driven by the younger cohorts, we next focused on gene expression signatures specific for these age groups. When we compare samples from aged and younger cohorts separately, we find 2106 probes significantly different between African Americans and Caucasians at FDR<5%, fold>1.2 in at least one cohort. The aged show 1469 and the younger 995 significant probes, respectively, with 356 overlapped probes (FDR<5% in both sets) with 1015 probes of the 2106 probes having significant nominal p<0.05 in both cohorts (Figure 5C). The list of genes is shown in Suppl. Fig. 3.

Of the 995 probes significantly different in young African Americans at baseline from young Caucasians, 410 are not different in aged African Americans (nominal p>0.05, or opposite fold change).

Several of the genes that differ in younger but not aged African Americans, such as guanylate binding proteins (GBP) 1 (−1.36) and 2 (−1.22), myxovirus resistance 2 (MX2, -1.26) and interferon regulatory factor 1 (IRF1, -1.29), are regulated by IFNs. Other are involved in B cell receptors signaling, such as B-cell scaffold protein with ankyrin repeats 1 (BANK1, 1.22), BCR downstream signaling 1 (BRDG1, 1.28), CD79B (1.31), SLAM family member 8 (SLAMF8, -1.2), T cell responses, such as IL-16 (−1.24), T cell receptor associated transmembrane adaptor 1 (TRAT1, 1.22), MHC class I polypeptide-related sequence A (MICA, -1.3), TAP binding protein (TAPBP, -1.25), perforin (PRF)1 (−1.43) or innate responses, such as TLR-10 (1.31). Differences are seen in transcripts of genes involved in lipid synthesis, i.e., acetyl-Coenzyme A carboxylase alpha (ACACA, -1.22), acyl-CoA thioesterase 11 (ACOT11, -1.23), cytochrome P450, family 2, subfamily S, polypeptide 1 (CYP2S1, -1.35), family 2, subfamily S, polypeptide 1) CYP2S1, -1.35), family 4, subfamily F, polypeptide 3 (CYP4F3, -1.37), lipid catabolism, such as peroxisome proliferator-activated receptor gamma, coactivator 1 beta (PPARGC1B, -1.22), aldehyde metabolism, i.e., aldehyde dehydrogenase 2 (ALDH2, -1.35) 3 family, member B1 (ALDH3B, -1.31), glycolysis, i.e., aldolase A, fructose-bisphosphate (ALDOA, -1.25), hexokinase (HK)3 (−1.2), hexokinase domain containing 1 (HKDC1, -1.3), pyruvate kinase, muscle (PKM)2 -1.2), pyruvate dehydrogensa kinase (PDK)3 (−1.23), pyruvate dehydrogenase phosphatase regulatory subunit (PDPR, -1.22) and the pentose phosphate pathway, i.e., glucose-6-phosphate dehydrogenase (G6PD, -1.26). Nine genes or pseudogenes encoding ribosomal proteins distinguish younger African Americans from younger Caucasians; 6 of those are higher in young African Americans.

Analysis by Ingenuity shows that cytotoxicity of lymphocytes and natural killer cells is reduced in African Americans as is T cell homeostasis, T cell development and differentiation of T helper lymphocytes (Figure 5D). The most significant difference in pathways is seen for antigen presentation, which is increased in younger African Americans. Regulators, which are reduced in younger African Americans, include regulation through CD3 and the T cell receptor as well as mTOR, which is downstream of the latter. In addition, regulation through CD40 ligand, IL-4, IFN-γ and insulin is reduced in younger African Americans.

Next, we tested the gene list for enrichment of genes specific to some blood cell type using IRIS dataset. We find that genes different between younger African Americans and younger Caucasians are enriched for B-cell specific genes (10 genes, 5.3 fold enrichment, p=3×10^−5^, 9 of 10 up-regulated in African Americans).

To test if the observed differences in gene expression profiles are caused by differences in circulating cell subsets we estimated proportions of different blood cells using CIBERSORT tool (17). We then compared African Americans vs. Caucasians within different age groups: only young, only aged and all samples. We did not find any consistent race-related differences after correction for multiple testing (data not shown).

## DISCUSSION

Previous studies have shown that ethnicity can influence immune responses to vaccination [1–6]. This is confirmed in our study, which shows higher antibody responses to the influenza A virus components of IIV3 or 4 in African Americans as compared to Caucasians. Previous studies largely focused on vaccines given to children that are immunologically naïve to the pathogen. In contrast, most adult individuals are immune to influenza virus, the focus of our study, either through previous infections or vaccinations. Influenza vaccines given to adults thus stimulate mainly recall responses, which can be combined with primary responses to new epitopes of the virus that were generated due to antigenic drifts. This primary response may be negligible as memory B cells to conserved epitopes dominate the response, a phenomenon referred to as antigenic sin [18].

In our study we tested antibody responses to the two influenza A virus strains of IIV3 or 4. The H1N1 virus evolved in 2009 when it caused a mild pandemic. Younger individuals were naïve to this virus, while approximately a third of the aged individuals had pre-existing antibodies. The 2009 H1N1 virus showed some similarities with the strain that caused the 1918 pandemic and then circulated in humans till 1957, which would explain cross-reactive antibody titers in some of the aged [19]. The H3N2 virus, which unlike H1N1 showed sufficient drift during our study period to warrant updating of the vaccine strain, has been circulating since 1968. Race-related differences in responses were seen for H1N1 but were less notable for H3N2 virus. This may reflect less frequent exposures to H1N1 virus, which has only been circulating sine 2009, as compared to H3N2 virus, which evolved more than 40 years ago and in turn could indicate that ethnicity-linked factors contribute less to B cell responses that have been repeatedly recalled than to primary responses or responses that had been initiated more recently.

Younger African Americans, who mounted higher neutralizing and IgG antibody responses compared to younger Caucasians, drove race-related differences in responses. One marked difference between younger Caucasians and younger African Americans is their influenza vaccination history; only 6 % (i.e., one individual) of the latter reported annual influenza vaccination as opposed to 42% of the former. Accordingly, baseline neutralizing antibody titers of young African Americans as compared to younger Caucasians were lower to H1N1 (median titers: 25 vs. 160) and H3N2 (median titers: 50 vs. 160). High titers of neutralizing antibodies can dampen stimulation of specific B cells by reducing the antigenic load [20]. and could thus explain lower responses in younger Caucasians. Nevertheless, when upon excluding young individuals, who reported that they either had never been vaccinated to influenza or had received the vaccine annually, significant differences were still seen between the remaining younger African Americans and Caucasians that stated occasional vaccinations (not shown). Other differences, such as higher numbers of circulating naïve B cells and lower expression of co-inhibitors on B cell subsets in younger African Americans or the very pronounced differences in the blood transciptome at baseline between the different cohorts, further suggest that differences in responses are caused by genetic factors or differences in lifestyle that are blunted upon aging.

Previous studies showed that subsets of circulating lymphocytes are predictive for immune responses following IIV3 vaccination [21]. In agreement, our results show that the younger African Americans with their higher frequencies of circulating B cell subsets mount higher responses compared to younger Caucasians.

Striking differences are observed in the blood transcriptome between African Americans and Caucasians. Some are conserved between the two age groups while others are only found between younger or aged groups. While previous studies assessed differences in gene expression between ethic groups, most of them focused on ethnic differences linked to diseases, such as cancer or metabolic disorders [22–24]. One study using B lymphoblastoids cells showed that ~83% of genes were differentially expressed among individuals, while only ~17% of genes were differentially expressed between populations of Caucasians and Africans [25]. Another study showed differential expression of 16% of all microRNAs between cohorts of different ethnicity [26]. None of these miRNAs that were significantly different are differentially expressed in our study. Two distinct miRNAs, miRMA182 and miR1974 show higher expression in African Americans regardless of age.

In our study differential gene expression between African Americans and Caucasians is in part influenced by age. African Americans show lower expression of myeloid genes indicative for reduced inflammatory responses regardless of age, while only the younger show increases in transcription of B cell-specific genes compatible with their higher antibody responses following vaccination. Of the B cell-specific genes 2 (B4GALT3 and FAM46C) are higher in African Americans regardless of age. KLF6, a tumor suppressor involved in B cell development and growth, is lower in younger African Americans. The others, BANK1, BRDG1, SPIB, STAP1, CD79B, FAM129C, and TLR10, all involved in B cell signaling, are higher in younger but not aged African Americans and may thus serve as predictors for responsiveness to IIV3 or 4 vaccination.

Cells of the adaptive immune system undergo metabolic reprogramming upon activation and this is essential for proliferation and the gain of effector functions [27, 28]. Upon stimulation mature naïve B cells proliferate and increase glycolysis as well as energy production through the tricarboxylic acid (TCA) cycle [28]. In addition, B cells catabolize amino acids. Once B cells cease to proliferate and transition into ASCs, which produce capacious amounts of immunoglobulins, glutamine becomes a primary carbon and energy source [29]. Upon stimulation mature naïve B cells differentiate either rapidly outside germinal centers into short lived plasma cells, which provide immediate protection through secretion of IgM, or they enter germinal centers where upon further signals from follicular T helper (T_FH_) cells they undergo class switching and affinity maturation before they differentiate into long-lived plasma cells or memory B cells [30]. The choice between class switch recombination (CSR) and plasma cell development (PCD) is driven by a set of mutually exclusive transcription factors [31] including Blimp1 (encoded by PRDM1) and Bach 2, which are influenced by the cells' metabolism [31]. As was shown through *in vitro* studies, activated B cells with increased mitochondrial mass, membrane potential and high levels of mitochondrial reactive oxygen species (mROS) undergo CSR unlike B cells with low mitochondrial mass and potential, which progress to PCD [32]. This in turn suggests that a shift in the balance of energy production through glycolysis or mitochondrial oxidative phosphorylation (OXPHOS) following the TCA cycle affects CSR, which is the desired outcome of vaccination. Our study shows that mitochondrial genes are overexpressed in African Americans and this is especially pronounced in the younger cohorts, while expression of differentially transcribed genes involved in glycolysis, i.e., ENO1, PKM and ALDOA or the glycolysis dependent pentose phosphate pathway, i.e., PGD, TKT, TALDO1, G6PD, RPIA is, but for the latter, lower in African Americans. This was linked to reduced mTOR signaling in African Americans, which is activated in T cells upon receptor signaling and promotes increased energy production through glycolysis by HIF-1α activity. Overall these results indicate that metabolic differences at baseline either caused by genetic factors or distinct lifestyles may affect B cell responses to IIV3 or 4 with cells of African Americans being more inclined to use OXPHOS while those from Caucasians appear to be more prone to use glycolysis.

One previous study focused on transcripts from adipose tissues to assess gene expression profiles linked to the increased insulin resistance in African Americans [33]; 49 of those same genes also differ in our analysis, which is a significant 3.01 fold enrichment (*p*-value = 6×10^−11^ by hypergeometrical test). With the caveat that lymphocytes are present in adipose tissue and may thus have contributed to the gene expression results of Das *et al*. [33], the significant overlap nevertheless indicates general race- or lifestyle-related differences.

In summary this study shows higher antibody responses to the H1N1 component of IIV3 or 4 in younger African Americans than younger Caucasians. This race-related difference is not observed in the aged. In addition, the blood transcriptome prior to vaccination shows marked race-related differences, which again are affected by age. One of the most striking differences in the increase in expression of B cell-related genes in younger African Americans, which may be candidates to predict high responsiveness to IIV3 or 4.

## MATERIALS AND METHODS

### Human subjects

Study subjects from the research triangle area (Durham-Raleigh-Chapel Hill) of North Carolina were recruited during the flu season over a period of 5 years (2011-2015) and consented at the Clinical Research Unit at Duke University Medical Center (Durham, NC, USA), in accordance with the Institutional Review Boards of both Duke University and the Wistar Institute (Philadelphia, PA, USA). Subjects were divided into two groups based on age. Young were 30-40 years of age and aged individuals were >65 years of age. Subjects were excluded if they had contraindication to influenza vaccine, alcohol or substance abuse, immune suppression resulting from disease (clinically active malignancy, HIV/AIDS, immune disorders) or immunomodulatory drug use (chemotherapy, corticosteroids), inter-current illness (urinary tract infection, respiratory tract infection), bed-ridden or homebound. We collected demographic data and medical history including diagnosis, medications, vaccinations to influenza or other diseases, and previous infections to influenza or influenza-like diseases during the last 5 years from the enrolled subjects. Seasonal trivalent or quadrivalent influenza vaccine (FLUARIX^®^, GlaxoSmithKline, Brentford, UK) was administered via intramuscular route in the deltoid muscle and blood was collected into heparinized tubes on days 0, 7 and 14 or 21 following vaccination. Blood was shipped overnight to The Wistar Institute, Philadelphia, PA for further processing.

### PBMC and serum isolation

1.5 mL of blood was centrifuged to isolate serum. Heparinized blood was used for PBMC isolation. PBMCs were isolated from whole blood by Ficoll-Paque Plus (GE Healthcare Biosciences, Piscataway Township, NJ) density gradient centrifugation. Briefly, blood was overlayed onto ficoll and spun for 30 minutes at 2000 rpm without brake. PBMCs were obtained after treating the layer with red blood cell lysis buffer (eBioscience, San Diego, CA) and washed in HBSS buffer.

### Virus strains and purification

Influenza virus A/California/7/2009 (H1N1) pdm09-like virus, A/Perth/16/2009 (H3N2)-like virus, A/Victoria/361/2011 (H3N2)-like virus, A/Texas/50/2012 (H3N2)-like virus and A/Switzerland/9715293/2013 (H3N2)-like virus strains were provided by Centers for Disease Control and Prevention (CDC), Atlanta, Georgia. Viruses were expanded in 10 day old specific pathogen free embryonated eggs for 2 days at 35°C. Allantoic fluid was harvested and pelleted at 20,000 rpm for 1 h at 4°C. The pellet was resuspended in PBS and further purified by ultracentrifugation through 10-55% sucrose density gradient at 25,000 rpm for 2 h. Mean tissue culture infective dose (TCID_50_) was determined by infecting the serially diluted virus on Madin-Darby Canine Kidney (MDCK) cells for 3 days and scored for cytopathic effects (CPE).

### Virus neutralizing antibody assay

Human sera was screened for neutralizing antibodies against influenza viruses as described previously [34]. Briefly, serum was heat inactivated at 56°C for 30 minutes and was serially two- fold diluted with MEM at an initial dilution of 1:10 in a 96 well plate. The diluted serum was incubated with equal volume of 100 TCID_50_ influenza virus strains at a final concentration for 1 hour at 37°C with 5% CO_2_. Following incubation, the serum-virus mixture was added to MDCK cells and further incubated at 37°C for 2 h. Cells were washed and incubated with MEM containing TPCK trypsin for 3 days. The neutralizing antibody titer was determined as the highest serum dilution in which 50% of MDCK cells were intact by scoring for cytopathic effect (CPE).

### ELISA

Nunc Maxisorp™ plate were coated overnight at 4°C with 10 μg/ml of purified influenza H1N1 or H3N2 virus along with isotype standards for IgA1, IgG and IgM (Athens Research & Technology, Inc., Georgia, USA). The plates were blocked with 3% BSA (Sigma, St. Louis, MO) in PBS for 2 h at room temperature. Plates were washed with PBS plus 0.01% Tween 20 (PBST) and incubated with serum samples) at a dilution of 1/250 for 2h. Following the incubation, the plates were washed 4 times with PBST and incubated with secondary alkaline phosphatase conjugated anti-human IgA1 at 1:1000, IgG at 1:3000 and IgM at 1:1000 dilution (SouthernBiotech, Alabama, USA) for 1 h. Subsequently, the plates were washed 4x with PBST and developed with pNPP (Sigma Aldrich, Missouri, USA) alkaline phosphatase substrate and read on a microplate reader at 405 nm. Antibody concentrations were determined based on the isotype standard curve and expressed in μg/ml values.

### Flow cytometry

Multiparametric flow cytometry was performed on young and aged PBMCs using a panel to detect B cell subsets by identifying mature naïve B cells (CD19^+^CD20^+^IgD^+^CD27^−^CD38^−^), transitional B cells (CD19^+^CD20^+^IgD^+^CD27^+/−^CD38^+/−^), non-switched memory B cells (CD19^+^CD20^+^IgD^+^CD27^+^CD38^−^), switched memory B cells (CD19^+^CD20^+/−^IgD^−^CD27^+^CD38^−^), double-negative B cells (CD19^+^CD20^+^IgD^−^CD27^−^CD38^−^) and antibody secreting cells (CD19^+^CD20^−^IgD^−^CD27^++^CD38^++^). PBMCs were stained first with antibody conjugates for the following surface markers: CD3-Pacific Blue (UCHT1, Biolegend, San Diego, CA), CD14-Pacific Blue (M5E2, Biolegend) as dump, CD19-BV650 (HIB19, Biolegend), CD20-BV570 (2H7, Biolegend), CD27-BV785 (O323, Biolegend), CD38-BV711 (HIT2, Biolegend), IgD-PerCP/Cy5.5 (IA6-2, Biolegend), BTLA-PE(MIH26, Biolegend), PD1-PE/Cy7 (EH12.2H7, Biolegend) LIVE/DEAD Fixable Aqua Dead Cell Stain (Life Technologies, Carlsbad, CA) for 30 min at 4°C. Cells were washed twice with cell staining buffer (Biolegend) and fixed/permiabilized using Cytofix/Cytoperm (BD Biosciences). Intracellular antibodies were detected by staining for IgG-BV605 (G18-145, BD Biosciences, San Jose, CA) and IgM-APC/Cy7 (MHM-88, Biolegend) for 30 min at 4°C. Cells were washed twice resuspended in BD stabilizing fixative until acquisition on a BD LSR II flow cytometer and analyzed using FlowJo (Tree Star, Ashland, OR).

The gating strategy is shown in Suppl. Figure 3.

### Microarray data

PAXgene tubes were stored at −80°C until RNA extraction. RNA was extracted using the PAXgene Blood RNA Kit IVD for isolation and purification of intracellular RNA from blood stabilized in PAXgene Blood RNA Tubes according to the manufacturers directions. RNA integrity was assessed using a bioanalyzer and only samples with an RNA integrity (RIN) # of >7.5 were processed for arrays. A constant amount (400 ng) of total RNA was amplified, as recommended by Illumina and hybridized to the Illumina H12-v4 human whole genome bead arrays.

Illumina GenomeStudio software was used to export expression levels and detection p-values for each probe of each sample. Signal intensity data was quantile normalized and log2 transformed. CIBERSORT software [17] was used to estimate proportions for different blood cell populations. Significance of expression level differences or cell proportion differences between any two groups baseline values or response values were done using linear regression with race and vaccination years as factors with correction for multiple testing to estimate False Discovery Rate (FDR) done according to Storey [35]. FDR < 5% was used as a significance threshold. Gene set enrichment analysis for biological functions, canonical pathways and upstream regulators was done using QIAGEN's Ingenuity^®^ Pathway Analysis software (IPA^®^, QIAGEN Redwood City, www.qiagen.com/ingenuity) and only results with predicted activated state (calculated by IPA activation Z-score of 2 or more) were considered. Pathways with FDR < 10% were also considered. Enrichment of cell type specific genes from IRIS database was done using Fisher exact test with FDR estimated by Benjamini-Hochberg procedure.

## SUPPLEMENTARY MATERIALS FIGURES AND TABLES




