# Supplementary material for: Race-related differences in antibody responses to the inactivated influenza vaccine are linked to distinct pre-vaccination gene expression profiles in blood
Source: Oncotarget. 2016 Aug 30;7(39):62898–911. doi: 10.18632/oncotarget.11704 (PMC5325335; doi:10.18632/oncotarget.11704)
Supplement: Supplementary file 1 [file oncotarget-07-62898-s001.pdf]

Race-related differences in antibody responses to the inactivated influenza vaccine are linked to distinct pre-vaccination gene expression profiles in blood

Supplementary Material

Fig. S1

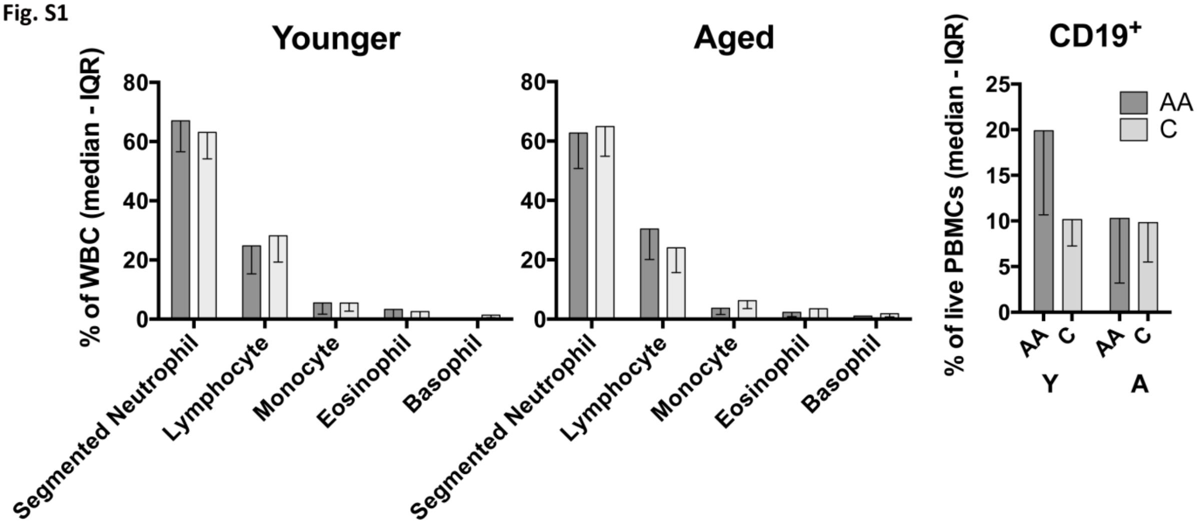

Suppl. Fig. 1. Distribution of cell subsets in blood.

Blood was analyzed for percentages of the indicated cell subsets. Graphs to the right and in the middle show results for young and aged African Americans (dark grey bars) and Caucasians (light grey bars). Number of samples AA, Y: 4, C, Y: 52; AA, A: 3, C, A: 63.

The graph on the left shows percentages of $CD19^{+}$cells over live lymphoid cells in the 4 cohorts. Number of samples AA, Y: 16, C, Y: 90; AA, A: 9, C, A: 145.

Fig. S2

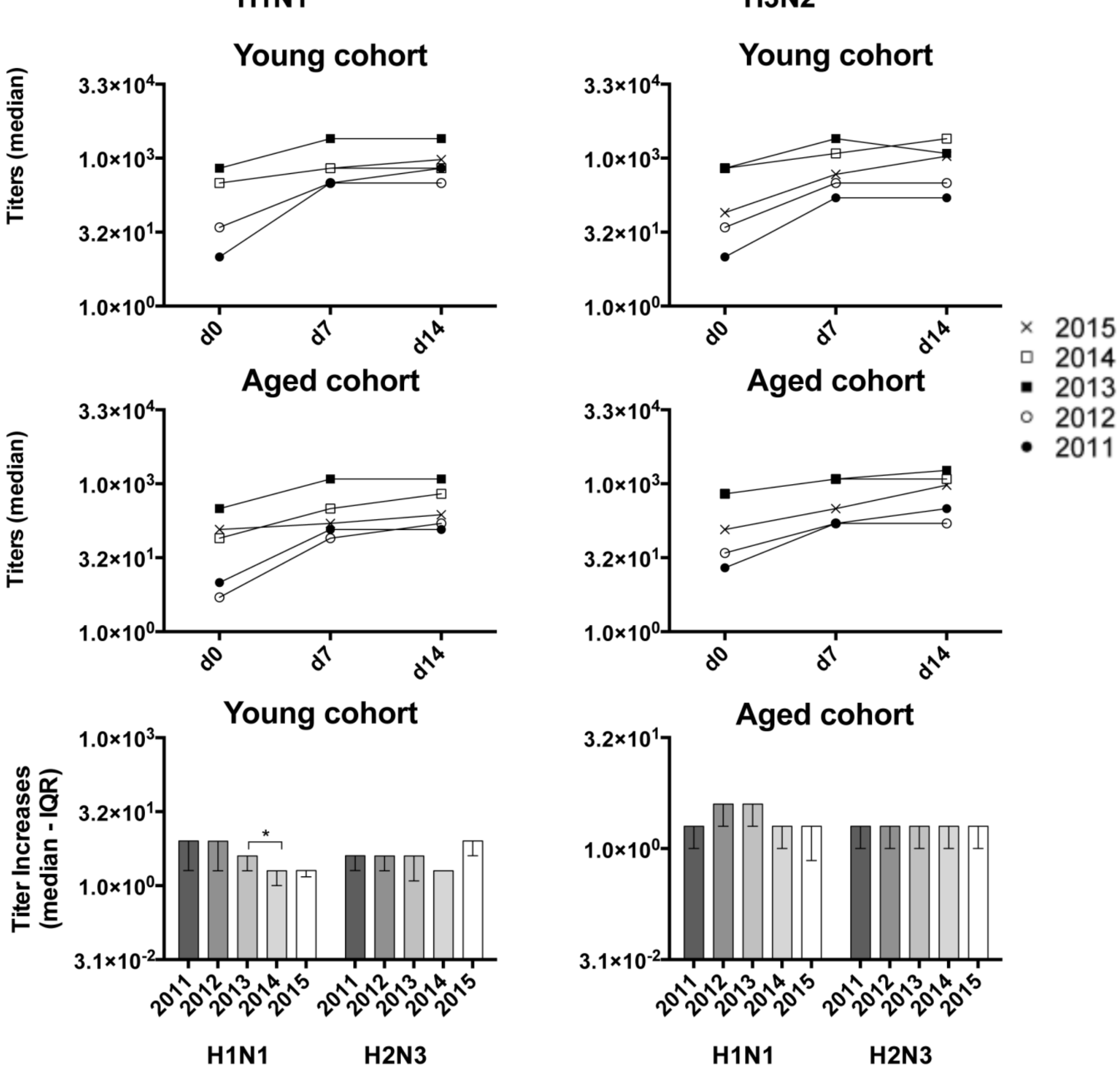

Suppl. Fig. 2

The upper and middle graphs show VNA titers at baseline and on days 7 and 14 or 21 after vaccination to H1N1 and H3N2 in young and aged individuals. Caucasians and African Americans were analyzed together. The following results showed significant differences by 2-way Anova: H1N1, Y d0: 2011-2012: $p = 0.04$, 2012 – 2013 $p = 0.007$; d 7 and d 14: 2013 to all years with p-values < 0.0001; H1N1, A, d7: 2011-2012: $p < 0.0001$, 2012-2013: $p < 0.001$, 2012-2014: $p = 0.3$, 2013-2014: $p = 0.002$, 2013-2015: $p < 0.0001$, d14: 2011-2013: $p<0.0001$, 2011-2014: $p = 0.03$, 2012-2013 $p < 0.0001$, 2012-2014: $p = 0.01$, 2013-2014: $p = 0.0002$,

2013-2015: p < 0.0001; H3N2, Y, d7: 2011-2013: p = 0.003, 2011-2014: p = 0.01, 2012-2013: p < 0.0001, 2012-2014: p = 0.003, 2013-2015: p = 0.002; d14: 2011-2013: p = 0.02, 2011-2014: p = 0.02, 2012-2013: p = 0.001, 2012-2014: p = 0.002; H3N2, A, d7: 2011-2013: p = 0.04, 2012-2013: p = 0.01; d14: 2011-2013: p = 0.01, 2012-2013 p = 0.0005, 2012-2015: p = 0.007.

The graphs at the bottom show titer increases to the different viruses according to study years. A line within the graph indicates the one significant difference in the younger.

Fig. S3

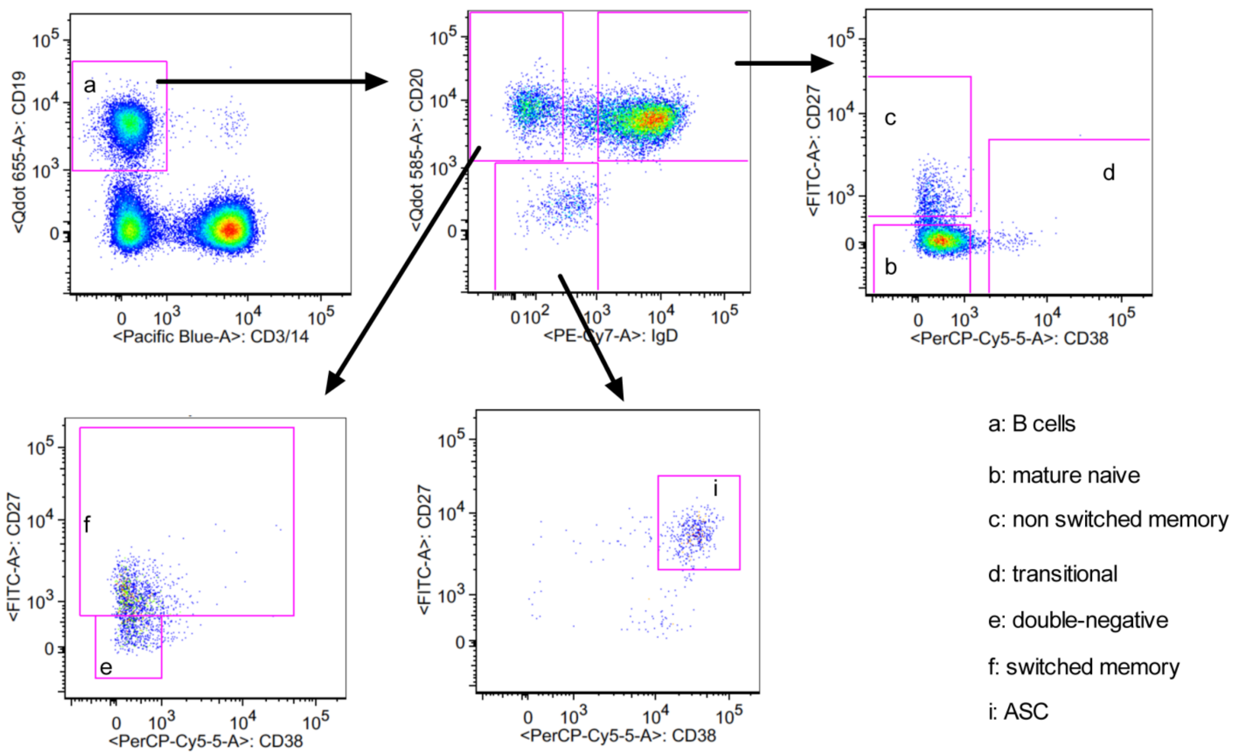

Suppl. Fig. 3 Gating Strategy

The graphs show our gating strategy for enumerating B cell subsets. Cells were initially gated on lymphoid cells, single cells and then live cells. These gates are not shown. The first graph in the left upper corner shows gating on $CD19^{+}CD3^{-}$ cells as our pan B cell gate. These cells were then gated on IgD and CD20. Double positive cells were gated on CD27 and CD38 to identify mature naïve, non-switched memory and transitional B cells. IgD-$CD20^{+}$ cells were gated on

CD27 and CD38 to distinguish double negative B cells from switched memory cells. IgD-CD20- cells were gated on $CD27^{hi}CD38^{hi}$ to identify antibody-secreting cells (ASC).

Suppl. Table 1. Enrollment table.

	Number of Tested Individuals				
**(A) Year of enrollment**	**2011**	**2012**	**2013**	**2014**	**2015**
**AA, Y**	6	6	2	2	1
**AA, A**	2	2	1	2	3
**C, Y**	9	21	25	25	14
**C, A**	26	33	33	32	27
**(B) Number of years tested**	**5x**	**4x**	**3x**	**2x**	**1x**
**AA, Y**	0	0	2	3	7
**AA, A**	1	0	0	1	3
**C, Y**	0	8	6	16	15
**C, A**	4	11	8	17	32

Suppl. Table 2. Genes significantly different between all or only young African Americans and Caucasians.

IRIS = shows 1 of the gene is from B or Myeloid cells as defined in IRIS dataset. set = shows 1 if the gene was a part of "Young only" or "All" gene sets. fold = African American/Caucasian gene expression fold change between African American vs Caucasian within Young populations. pv = nominal p-value for comparisons within Young, Aged and ALL (combined) populations between African American/Caucasian. FDR = false discover rate for comparisons within Young, Aged and ALL (combined) populations between African American/Caucasian.
