# Supplementary material for: Race-related differences in antibody responses to the inactivated influenza vaccine are linked to distinct pre-vaccination gene expression profiles in blood
Source: Oncotarget. 2016 Aug 30;7(39):62898–911. doi: 10.18632/oncotarget.11704 (PMC5325335; doi:10.18632/oncotarget.11704)
Supplement: Supplementary file 2 [file oncotarget-07-62898-s002.pdf]

Supplementary table 1. Genes sginficantly different between all or only young African Americans and Caucasians.

IRIS = shows 1 of the gene is from B or Myeloid cells as defined in IRIS dataset.
set = shows 1 if the gene was a part of "Young only" or "All" gene sets
fold = African American/Caucasian gene expression fold change between African American vs Caucasian within Young populations
pv = nominal p-value for comparisons within Young, Aged and ALL (combined) populations between African American/Caucasian
FDR = false discover rate for comparisons within Young, Aged and ALL (combined) populations between African American/Caucasian

ID	Accession	Desciption	Symbol	Entrez	set Young unique	set ALL	IRIS B Cell	IRIS Myeloid	fold AA/C	pv Young	pv Aged	pv ALL	FDR Young	FDR Aged	FDR ALL
ILMN_2038775	NM_00106	tubulin, bet	TUBB2A	7280	0	1	0	0	2.79	2.5E-05	5.9E-12	2.2E-13	0%	0%	[illegible]
ILMN_1716733	NM_003970	myomesin	MYOM2	9172	0	1	0	0	3.00	9.7E-05	9.7E-10	5.1E-13	1%	0%	[illegible]
ILMN_1749834	XM_932890	PREDICTE	LOC388588	388588	0	1	0	0	4.53	5.4E-08	1.5E-06	8.2E-12	0%	0%	[illegible]
ILMN_1713458	NM_005332	hemoglobin	HBZ	3050	0	1	0	0	2.09	########	########	3.2E-05	14%	1%	[illegible]
ILMN_1652431	NM_001738	carbonic an	CA1	759	0	1	0	0	2.18	########	3.6E-06	2.4E-06	4%	0%	[illegible]
ILMN_1735712	NM_006121	keratin 1 (K	KRT1	3848	0	1	0	0	1.81	########	6.6E-05	8.3E-05	14%	0%	[illegible]
ILMN_2294762	NM_004038	amylase, al	AMY1A	276	0	1	0	0	1.28	########	4.5E-14	8.6E-10	19%	0%	[illegible]
ILMN_1697499	NM_002125	major histoc	HLA-DRB5	3127	1	1	0	0	5.79	########	0.1279	########	4%	36%	[illegible]
ILMN_3305475	XM_001725	PREDICTE	LOC729708	729708	0	1	0	0	3.95	1.8E-25	9.1E-16	9.9E-41	0%	0%	[illegible]
ILMN_1680652	NM_003944	selenium bi	SELENBP1	8991	0	1	0	0	1.87	########	7.9E-07	3.9E-07	4%	0%	[illegible]
ILMN_1774091	NM_000140	ferrochelata	FECH	2235	0	1	0	0	1.56	########	9.1E-06	5.8E-05	17%	0%	[illegible]
ILMN_2352921	NM_001724	2,3-bisphos	BPGM	669	0	1	0	0	1.93	########	4.9E-07	6E-09	1%	0%	[illegible]
ILMN_1750100	NM_020040	tubulin, beta	TUBB4Q	56604	0	1	0	0	1.52	8.6E-05	6.8E-15	1.1E-14	1%	0%	[illegible]
ILMN_1905548	CR627122	mRNA; cDN	NaN	na	0	1	0	0	1.66	########	3.6E-07	1.5E-07	4%	0%	[illegible]
ILMN_1766165	NM_000345	synuclein, a	SNCA	6622	0	1	0	0	2.09	########	4.3E-06	3.7E-08	1%	0%	[illegible]
ILMN_1708323	NM_001037	aminolevuli	ALAS2	212	0	1	0	0	2.33	########	########	1.9E-05	2%	1%	[illegible]
ILMN_1713266	NM_017702	family with	FAM46C	54855	0	1	1	0	1.78	########	1.9E-05	3E-06	5%	0%	[illegible]
ILMN_1736911	NM_003275	tropomoduli	TMOD1	7111	0	1	0	0	1.47	########	1.5E-06	1.1E-05	16%	0%	[illegible]
ILMN_1808456	NM_025057	chromosom	C14orf45	80127	0	1	0	0	1.62	########	4.8E-09	1E-08	3%	0%	[illegible]
ILMN_1678919	NM_018566	YOD1 OTU	YOD1	55432	0	1	0	0	1.68	########	2.5E-06	1.3E-07	3%	0%	[illegible]
ILMN_2231051	NM_152773	t-complex 1	TCP11L2	255394	0	1	0	0	1.34	########	3.3E-14	4E-13	2%	0%	[illegible]
ILMN_1781966	NM_030758	oxysterol bi	OSBP2	23762	0	1	0	0	1.31	0.192	4.3E-05	########	40%	0%	[illegible]
ILMN_1724437	NM_014291	glycine C-a	GCAT	23464	0	1	0	0	1.21	0.1309	1.5E-08	7.8E-06	34%	0%	[illegible]
ILMN_1695187	NM_002102	glycophorin	GYPE	2996	0	1	0	0	1.67	########	0.00011	3E-05	9%	1%	[illegible]
ILMN_1694027	NM_144665	sestrin 3 (S	SESN3	143686	0	1	0	0	1.83	8.7E-05	5.5E-07	5.8E-09	1%	0%	[illegible]
ILMN_1720373	NM_003486	solute carri	SLC7A5	8140	0	1	0	0	1.39	########	6.7E-10	6.6E-10	2%	0%	[illegible]
ILMN_2152465	NM_005028	phosphatid	PIP5K2A	5305	0	1	0	0	1.71	########	8.3E-08	3.6E-07	3%	0%	[illegible]
ILMN_1759117	NM_021083	X-linked Kx	XK	7504	0	1	0	0	1.52	########	2E-06	1.5E-06	6%	0%	[illegible]
ILMN_1705458	NM_015431	tripartite mo	TRIM58	25893	0	1	0	0	1.82	########	4.5E-05	1.4E-06	3%	0%	[illegible]
ILMN_1696512	NM_016633	alpha hemo	AHSP	51327	0	1	0	0	2.20	8.4E-06	2E-05	7.1E-08	0%	0%	[illegible]
[illegible]	[illegible]	[illegible]	[illegible]	[illegible]	[illegible]	[illegible]	[illegible]	[illegible]	[illegible]	[illegible]	[illegible]	[illegible]	[illegible]	[illegible]	[illegible]
